# Targeting Trachoma Control through Risk Mapping: The Example of Southern Sudan

**DOI:** 10.1371/journal.pntd.0000799

**Published:** 2010-08-17

**Authors:** Archie C. A. Clements, Lucia W. Kur, Gideon Gatpan, Jeremiah M. Ngondi, Paul M. Emerson, Mounir Lado, Anthony Sabasio, Jan H. Kolaczinski

**Affiliations:** 1 School of Population Health, University of Queensland, Herston, Queensland, Australia; 2 Australian Centre for International and Tropical Health, Queensland Institute of Medical Research, Brisbane, Queensland, Australia; 3 Ministry of Health, Government of Southern Sudan, Juba, Southern Sudan; 4 The Carter Center, Juba, Southern Sudan; 5 Department of Public Heath and Primary Care, University of Cambridge, Cambridge, United Kingdom; 6 The Carter Center, Atlanta, Georgia, United States of America; 7 Malaria Consortium – Southern Sudan Office, Juba, Southern Sudan; 8 Malaria Consortium – Africa Regional Office, Kampala, Uganda; 9 Disease Control and Vector Biology Unit, London School of Hygiene and Tropical Medicine, London, United Kingdom; University of Tennessee, United States of America

## Abstract

**Background:**

Trachoma is a major cause of blindness in Southern Sudan. Its distribution has only been partially established and many communities in need of intervention have therefore not been identified or targeted. The present study aimed to develop a tool to improve targeting of survey and control activities.

**Methods/Principal Findings:**

A national trachoma risk map was developed using Bayesian geostatistics models, incorporating trachoma prevalence data from 112 geo-referenced communities surveyed between 2001 and 2009. Logistic regression models were developed using active trachoma (trachomatous inflammation follicular and/or trachomatous inflammation intense) in 6345 children aged 1–9 years as the outcome, and incorporating fixed effects for age, long-term average rainfall (interpolated from weather station data) and land cover (i.e. vegetation type, derived from satellite remote sensing), as well as geostatistical random effects describing spatial clustering of trachoma. The model predicted the west of the country to be at no or low trachoma risk. Trachoma clusters in the central, northern and eastern areas had a radius of 8 km after accounting for the fixed effects.

**Conclusion:**

In Southern Sudan, large-scale spatial variation in the risk of active trachoma infection is associated with aridity. Spatial prediction has identified likely high-risk areas to be prioritized for more data collection, potentially to be followed by intervention.

## Author Summary

Trachoma, caused by the bacterium *Chlamydia trachomatis*, is the leading cause of preventable blindness worldwide and a major cause of blindness in Southern Sudan. However, the trachoma distribution in Southern Sudan has only been partially established and many communities in need of intervention have not been identified or targeted. Incomplete mapping and intervention coverage is largely attributable to trachoma resources being scarce and not always deployed most efficiently. The present study aimed at improving programme efficiency by developing maps to help target the available resources for trachoma surveys and interventions to areas where these are most needed. Data on active trachoma prevalence, collected during baseline surveys between 2001 and 2009, were incorporated into Bayesian geostatistical models to develop a national trachoma risk map. The model predicted the west of the country to be largely at no or very low trachoma risk, while most of the high-risk areas are located in the centre, north, and south-east. Risk mapping has allowed Southern Sudan's trachoma control programme to identify areas where collection of additional data would be most useful. As a direct result, baseline data were collected in March 2010 for the whole of Unity State, with antibiotic mass drug administration being scaled up from June 2010 onwards.

## Introduction

Trachoma, caused by the bacterium *Chlamydia trachomatis*, is the most common infectious cause of blindness and the leading cause of preventable blindness worldwide [1], [2]. The disease is easily transmitted through transfer of ocular secretions infected with *C. trachomatis* to the eyes of an uninfected individual by flies, hands, towels or sharing of other personal items. Repeated infection with *C. trachomatis* leads to scarring of the conjunctiva and eventually entropion, causing the lashes of the inwardly-turned eyelid to abrade the corneal surface, a condition referred to as trichiasis [3], [4]. Unless eyelid deformation is managed surgically, trichiasis causes irreversible scarring of the cornea leading to corneal opacity and, eventually, blindness. Trachomatous trichiasis (TT) in children is an indication of high-intensity transmission.

Like all other neglected tropical diseases (NTDs) trachoma is associated with poverty [5], [6], as well as poor hygiene [7], [8]. Prevention is partly based on improving personal hygiene by promoting facial cleanliness and providing clean water for face washing, and promoting the safe disposal of human faeces, thereby reducing fly abundance [9]. **F**acial cleanliness and **E**nvironmental improvement form two of the four components of the World Health Organization (WHO) recommended “SAFE” strategy for trachoma control, which also includes **S**urgical correction of trichiasis and mass drug administration (MDA) of **A**ntibiotics in endemic communities [10].

Studies have shown trachoma risk to be associated with attributes of the physical and social environment [8]. Risk factors include environmental aridity, nomadic pastoral livelihoods (i.e. predominantly livestock-rearing), increasing distance from water sources and household crowding [7], [11]–[13]. Given that environmental factors are important drivers of trachoma risk, it is plausible to predict the spatial distribution of trachoma using statistical associations between disease prevalence and environmental variables. Linkage of trachoma survey data to environmental variables can be performed in a geographical information system (GIS). Statistical models can then be used to estimate the relationship between trachoma risk and environmental variables, and to predict trachoma risk in non-sampled locations based on their environmental attributes. Schemann and colleagues used such (non-spatial) multivariate logistic regression model with trachoma data from Mali, finding that prevalence of active trachoma was negatively correlated with rainfall, in turn resulting in a north-south gradient of trachoma risk [14].

A major recent advance in risk mapping has been the development of model-based geostatistics, providing a statistically robust platform for prediction of disease risk based simultaneously on environmental covariates and functions of spatial autocorrelation [15]. The model outputs are distributions, rather than point estimates, which fully represent prediction uncertainties and enable flexible statistical inference, such as determining the probability that risk in a location is above a specific threshold [16]. Risk maps derived from model-based geostatistical predictions have been used to increase the efficiency of some NTD control programmes, such as for schistosomiasis and soil-transmitted helminths in sub-Saharan Africa, by allowing targeting of resources to areas where they were likely to have the greatest impact [16]–[23]. However, to date these epidemiological advances have not been applied to the management of trachoma control programmes.

Cataract and trachoma are the two most important causes of blindness in Southern Sudan [24]. Recent surveys have found both extremely high prevalence of active trachoma (trachomatous inflammation-follicular (TF) and/or trachomatous inflammation-intense (TI)) and evidence of TT in children in some of the areas surveyed [25], [26]. These findings indicate that trachoma constitutes a major problem to public health in Southern Sudan [27]. However, not all of Southern Sudan is equally at risk, as indicated by recent surveys that identified areas where trachoma is not endemic [28]. Generating a better understanding of the geographical distribution of trachoma is therefore important so that the limited available resources can be better targeted. To provide the National Trachoma Control Programme with a tool to prioritise areas for SAFE intervention we develop a model that takes account of spatial correlation in the data, aiming to identify important environmental predictors of trachoma risk in Southern Sudan and to use these to develop a trachoma risk map.

## Methods

### Ethics Statement

The risk mapping analysis received ethical approval from the Directorate of Research, Planning and Health System Development, Ministry of Health, Government of Southern Sudan (MoH-GoSS). The study consisted entirely of secondary analysis of data from population-based prevalence surveys (PBPS) for which separate ethical approval had been obtained from the same institutional review board.

### Trachoma Surveys

Field survey data were obtained from PBPS conducted and previously reported by The Carter Center in Unity [26], [29], Jonglei [25], Eastern Equatoria, Central Equatoria and Upper Nile States [30], and by Malaria Consortium and the MoH-GoSS, in Western Equatoria State [28]. All PBPS used a two-stage cluster design with randomised selection of communities and individuals within communities. Details on the survey design and ethical approval are provided elsewhere [25], [28], [31].

Diagnosis of trachoma was based on physical examination of the conjunctiva of the survey participants by trained personnel and the stage of trachoma was graded using the simplified WHO scheme [32]. In the current study, only data on active trachoma from children aged 1–9 years were included because trachoma in this age group most likely reflected local transmission. Presence of trachomatous inflammation (either TF or TI) of the conjunctivae of one or both eyes was considered a positive diagnosis of active trachoma. The age and sex of the participants were recorded during each of the surveys. The field survey locations were geo-referenced using a global positioning system, or by matching community names with those in an existing geo-referenced community database compiled by the Southern Sudan Guinea Worm Eradication Program. The final dataset contained data collected between 2001 and 2009 from 6345 children aged 1–9 years in 112 communities that we were able to geo-locate. The dataset included 3181 boys and 3164 girls.

### Environmental Variables

**Figure 1:**
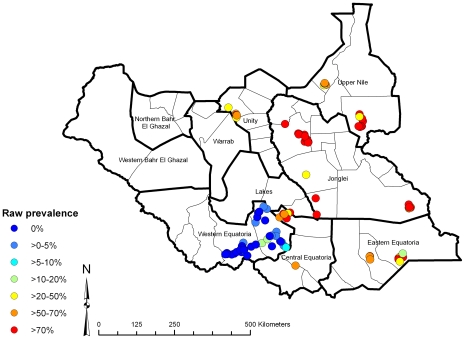
Observed prevalence of active trachoma using data from population-based prevalence surveys, in children aged 1–9 years, Southern Sudan, 2001–2009.

The trachoma field survey data were plotted in the GIS software ArcView (Version 9.2, ESRI, Redlands, California, USA) (Figure 1). Digital information on environmental variables was obtained from different sources. Elevation above mean sea level and interpolated long-term average monthly minimum and maximum land surface temperature and rainfall were obtained from the WorldClim project (www.worldclim.org). Minimum, maximum and mean normalised difference vegetation index (NDVI) and land surface temperature (LST) for 1982–1998 were obtained from the National Oceanographic and Atmospheric Administration's (NOAA) Advanced Very High Radiometer (AVHRR). Classified land cover variables were obtained from the International Geosphere-Biosphere Programme (IGBP) (http://www.igbp.net, derived from AVHRR data), grouped into wooded savannah, savannah, cropland/shrubland/grassland and forest/wetland, and from the United States Geological Survey global land cover database (http://edc2.usgs.gov/glcc/glcc.php). The location of perennial inland water bodies was provided by the Food and Agriculture Organization of the United Nations and used to calculate the distance of survey locations from permanent water sources. These variables were linked in ArcView to the trachoma field data according to location.

### Statistical Analysis

Co-linearity in the continuous environmental variables was assessed using Pearson's correlation coefficients and for all pairs of variables with correlation >0.7, the variable with the highest *p*-value in bivariate logistic regression models (with trachoma prevalence as the outcome) was excluded. Variance inflation factors (VIF) were also examined and variables with a VIF >10 were removed. Environmental variables were selected using backwards stepwise logistic regression in Stata (Version 10, Statacorp, College Station, Texas, USA) using an exit criterion of Wald's p>0.1 and an entry criterion of Wald's p≤0.05. Selected environmental variables included long-term average annual rainfall (continuous in mm) and IGBP land cover (categorical). Age (in years) and sex of survey participants were retained in the models as individual-level covariates.

Logistic regression models were developed in the freely available Bayesian statistical software WinBUGS version 1.4 (Medical Research Council Biostatistics Unit, Cambridge, UK/Imperial College London, London, UK). These models had the disease status (positive or negative) for active trachoma (TF and/or TI) in each child aged 1–9 years as the Bernoulli-distributed outcome (where positive  = 1 and negative  = 0). Two models were developed with the following parameters: model 1 had fixed effects for age, sex, long-term average annual rainfall and land cover and model 2, constructed using the principle of model-based geostatistics[15], had fixed effects for age, sex, long-term average annual rainfall and land cover plus geostatistical location-level random effects with a correlation structure defined by an isotropic exponentially decaying autocorrelation function. In this model, the environmental fixed effects are useful for explaining large-scale spatial variation (i.e. trend); and for spatial prediction, which is based both on the environmental attributes of the prediction locations and observed prevalence at nearby survey locations (captured by the geostatistical random effect). The individual fixed effects are useful for adjusting the model estimates for any age or sex differences between the survey locations. These models were constructed separately to determine whether the inclusion of the geostatistical component improved the predictive ability of the model. All model parameters were given non-informative prior distributions.

Model selection was based on the deviance information criterion (DIC, a Bayesian analogue of Akiake's information criterion, for which a lower value of the DIC indicates a more favourable compromise between model fit and parsimony). Spatial prediction based on model 2 was done in WinBUGS by combining kriging of the random effects (i.e. estimating their values at non-sampled locations using this geostatistical smoothing method [33]) with application of the coefficients of the community-level environmental covariates to the values of these covariates at all non-sampled locations. Predictions were thus based on the environmental covariates and the geostatistical random effects.

Spatial predictions were validated by randomly partitioning the survey locations into four approximately equal-sized subsets of survey locations. The model was built using three subsets and was used to predict prevalence of active trachoma for individuals at the locations of the fourth subset. This procedure was repeated four times, each time predicting prevalence of trachoma at the locations of a different subset. Thus, predicted prevalence values were obtained for all 112 locations.

Discriminatory performance was assessed at the individual level and at the location level. For the former, the individual's predicted risk of trachoma was compared to their observed trachoma status. For the latter, predicted prevalence was compared to observed prevalence dichotomised using the following thresholds: >0%, 10%, 40% and 70%. For each comparison, sensitivity of the predicted value was plotted against one minus the specificity (the receiver operating characteristic; ROC) and the area under the ROC was calculated. This was calculated separately for each subset, and for the pooled values from all four subsets. Values of area under the ROC ≥0.9 indicate excellent model discrimination, ≥0.7–0.9 indicate moderate model discrimination and <0.7 indicate poor model discrimination. Mean prediction error and mean absolute prediction error were also calculated to determine model calibration.

Isotropic semivariograms (i.e. semivariograms that did not vary by direction) were developed using the geoR library of the R statistical software package (Version 2.9.0, The R Foundation for Statistical Computing) to test spatial autocorrelation in the raw prevalence data and in the Pearson's residuals of models 1 and 2.

## Results

Prevalence of active trachoma in children aged 1–9 years was 48.2%, but this varied markedly between states of Southern Sudan, ranging from 2.2% to 77.6% (Table 1). No statistically significant difference was found in active trachoma prevalence between boys (47.3%) and girls (49.1%), but there was a significant negative correlation between active trachoma prevalence and age (Table 2).

**Table 1:** Descriptive statistics of active trachoma in children aged 1–9 years in geo-referenced communities.

STATE	Number of communities	Number TF or TI positive/Number screened (% positive)
Central Equatoria	9	324/464 (69.8)
Eastern Equatoria	9	437/686 (63.7)
Jonglei	29	1306/1713 (76.2)
Unity	12	484/863 (56.1)
Upper Nile	13	463/597 (77.6)
Western Equatoria	40	45/2022 (2.2)
Total	112	3059/6345 (48.2)

**Table 2:** Logistic regression models for active trachoma in children aged 1–9 years.

Variable	Model 1 OR (95% CI)	Model 2 OR (95% CI)
Age (years)	0.93 (0.91–0.95)*	0.90 (0.87–0.93)*
Sex: Female	1.05 (0.93–1.17)	1.01 (0.87–1.16)
Rainfall (per 100 ml)	0.55 (0.49–0.62)*	0.21 (0.08–0.46)*
Land cover: Savanna	1.77 (1.48–2.11)*	3.44 (0.50–13.98)
Land cover: Forest or wetland	1.20 (0.98–1.46)	2.48 (0.21–12.14)
Land cover: Grass, shrub, cropland	0.62 (0.50–0.75)*	0.81 (0.09–3.65)
Intercept (*β*)	−0.01 (−0.17–0.14)	−0.86 (−1.78–0.06)
ϕ	–	41.90 (6.05–172.90)
σ^2^	–	5.30 (3.23–8.86)
DIC	6554.9	4753.2

Reference sex is male; reference land cover is wooded savanna;

ϕ =  rate of decay of spatial correlation;

σ^2^ =  variance of spatial random effect;

*Significant at 5% level.

In the study communities, the average long-term average rainfall was 979 mm (range, 509–1470 mm). In both models there was a significant negative correlation between rainfall and the prevalence of active trachoma (e.g., model 2: OR 0.21, 95% CI 0.08–0.46, indicative of a 79% decrease in prevalence for a 100 mm increase in rainfall). Land cover was a significant explanatory variable in model 1, but not model 2.

**Figure 2:**
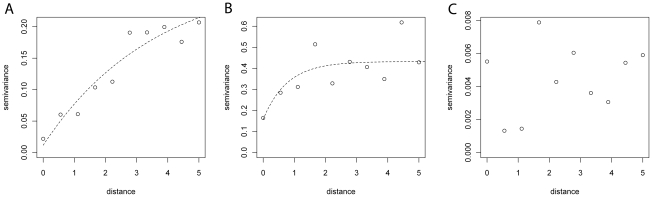
Semivariograms related to risk mapping models for active trachoma in children aged 1–9 years, Southern Sudan, 2001–2009. A) raw data and Person's residuals of: B) model 1; and C) model 2. Models 1 and 2 refer to the models presented in Table 1.

The unbounded semivariogram for the raw trachoma prevalence (Figure 2A) suggests a spatial trend. By contrast, the semivariogram of the Pearson's residuals of model 1 (Figure 2B) demonstrated second-order spatial autocorrelation (i.e. local clustering). The semivariograms of the Pearson's residuals of model 2 (Figure 2C) did not show spatial autocorrelation. In this model, the range of spatial autocorrelation can be calculated by 3/ϕ and is thus 0.07 decimal degrees (approximately 8 km). This value is indicative of the radius of trachoma clusters, as it represents the separating distance between two points at which spatial autocorrelation is <5%.

**Figure 3:**
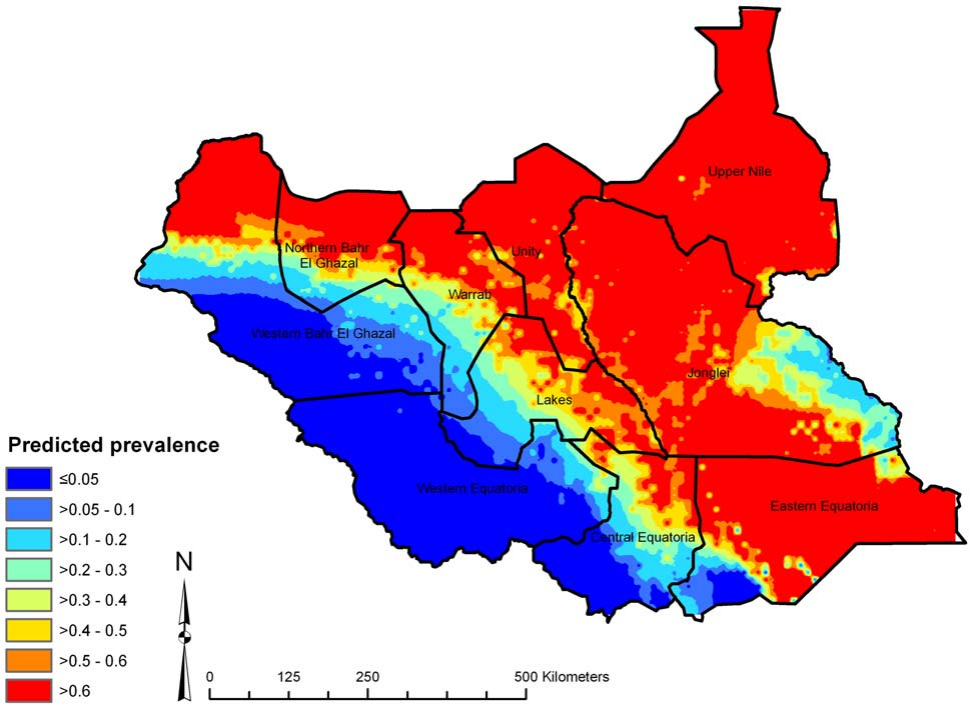
The median of the posterior distribution for predicted prevalence of active trachoma in children aged 1–9 years, Southern Sudan, 2001–2009.

**Figure 4:**
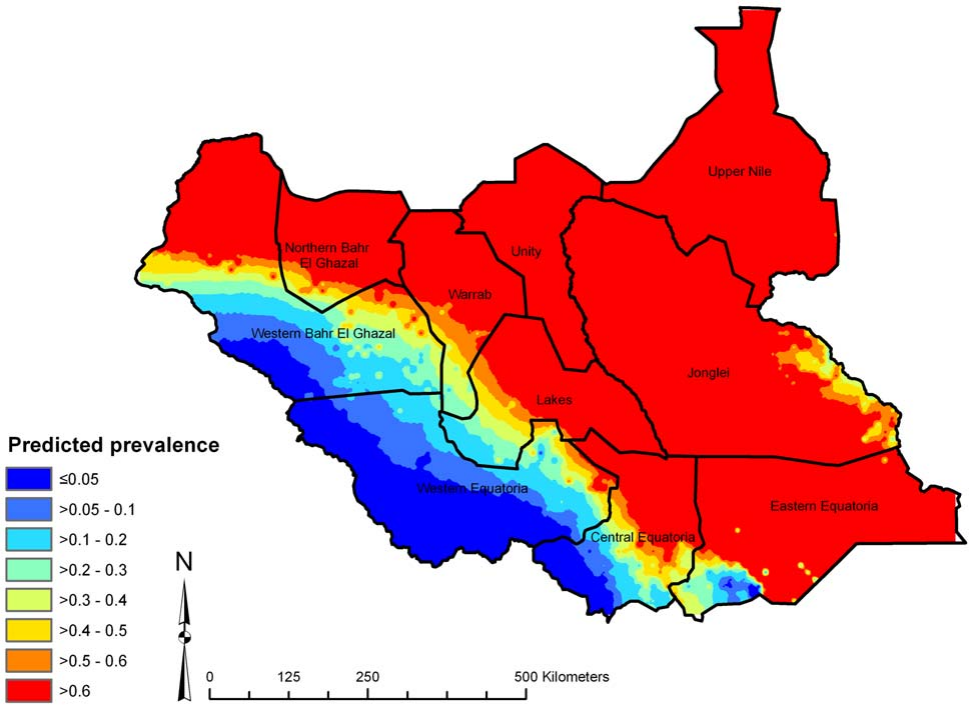
Upper quartile of posterior distribution of predicted prevalence of active trachoma in children aged 1–9 years, Southern Sudan, 2001–2009.

**Figure 5:**
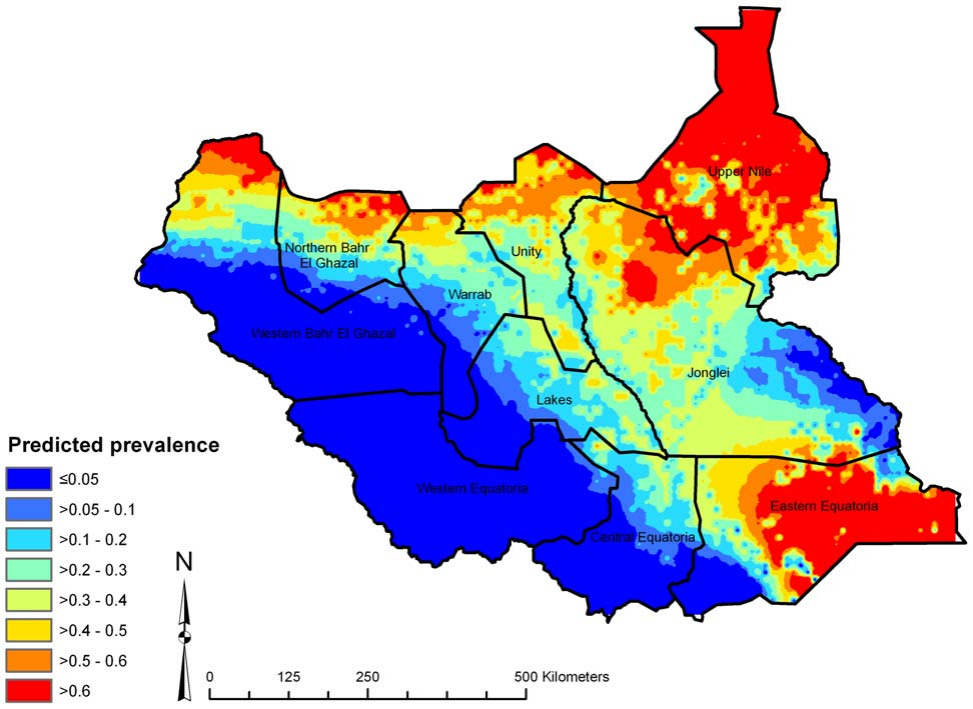
Lower quartile of posterior distribution of predicted prevalence of active trachoma in children aged 1–9 years, Southern Sudan, 2001–2009.

Here we present spatial predictions based on model 2, which had the lowest DIC. The map of the posterior median predicted prevalence of active trachoma (Figure 3) shows high predicted prevalence throughout central, northern and south-eastern Southern Sudan. Low predicted prevalence was apparent in the south-west, which were generally areas with higher long-term average rainfall. Examination of the upper and lower quartiles of the posterior distributions of predicted prevalence (Figures 4 and 5) suggest that large parts of Upper Nile, Unity, Jonglei and Eastern Equatoria States have a high probability of being endemic for trachoma, while large areas in the west of the country (particularly Western Equatoria State, the south-western part of Central Equatoria State and the southern part of Western Bar-el-Ghazal State) are unlikely to be at risk of trachoma. We can be particularly confident of the low predicted prevalence values in these latter states because of the low prediction standard errors.

**Figure 6:**
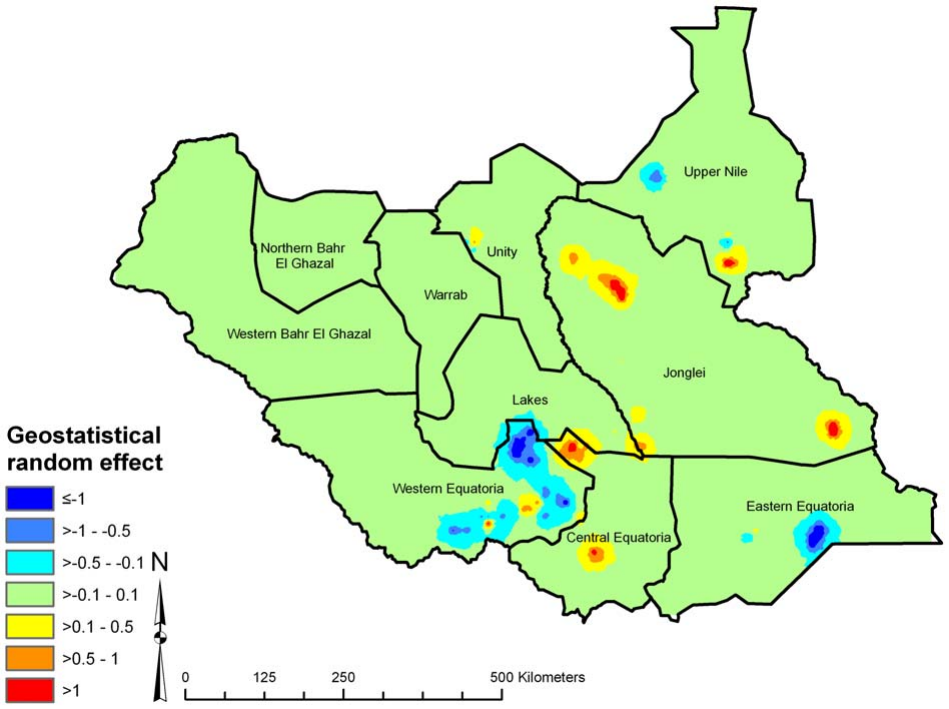
Spatially structured residual variation in prevalence of active trachoma in children aged 1–9 years, Southern Sudan, 2001–2009, after accounting for rainfall, land cover and age and sex of survey participants. Estimates were derived using a geostatistical random effect.

**Figure 7:**
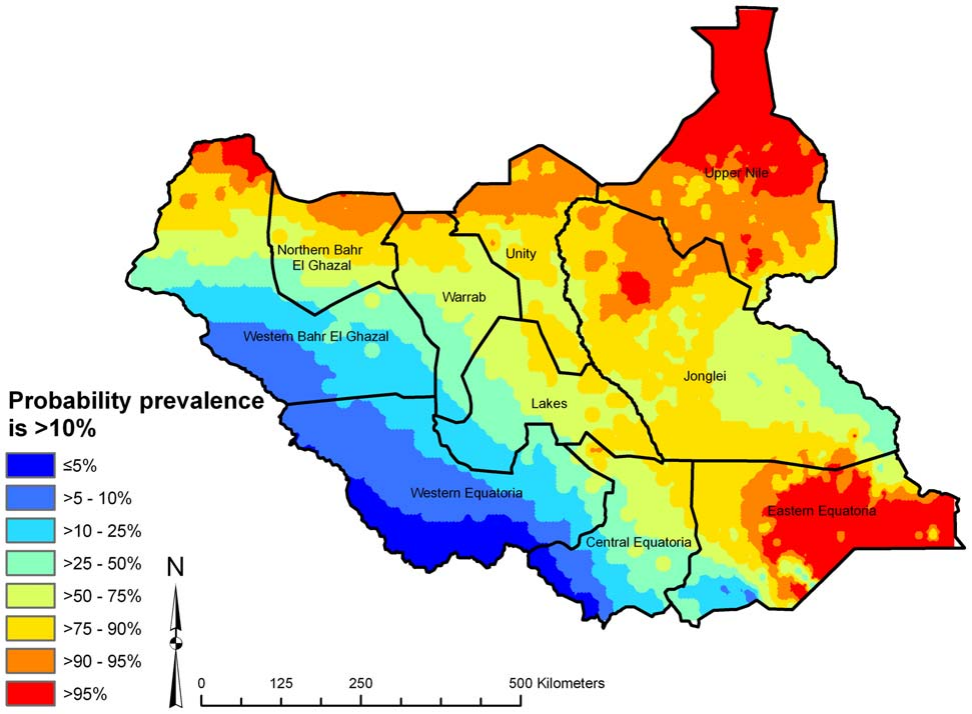
Predicted probability that prevalence of active trachoma is >10% in children aged 1–9 years, Southern Sudan, 2001–2009. Estimates were obtained from posterior predictive distributions derived using model-based geostatistics.

A map of the geostatistical random effects (Figure 6) suggests areas of high residual risk of active trachoma (after accounting for the fixed effects, rainfall, land cover, age and sex) in Upper Nile, Jonglei, Unity and Central and Eastern Equatoria States, and areas of low residual risk in Western and Eastern Equatoria and Northern Bahr-el-Ghazal States. From the posterior distributions of predicted prevalence, we also determined the probability that predicted prevalence of active trachoma was >10% (Figure 7), an indication as to whether antibiotic MDA is required – actual MDA decisions are based on prevalence of only TF, not TF plus TI, in children age 1–9 years as determined through PBPS [9]. Nevertheless, our probability map indicates that prevalence of active trachoma in much of south-western Southern Sudan is likely to be below the MDA intervention threshold.

Validation analysis of model 2 (Table 3) at the individual level found, pooled across all subsets, an area under the ROC of 0.80 (95% CI 0.79, 0.81), indicating good discriminatory performance of the model for an individual's probability of having active trachoma. At the location level, model 2 had excellent predictive ability to discriminate prevalence of active trachoma relative to thresholds of 0%, 10%, 40% and 70%, with areas under the ROC of 0.96 (95% CI 0.93, 0.99), 0.96 (95% CI 0.93, 1.00), 0.92 (95% CI 0.87, 0.98) and 0.80 (95% CI 0.72, 0.88) respectively (pooled across all subsets). Mean prediction error was −0.012 and mean absolute prediction error was 0.170, indicating that, on average, the model under-predicted prevalence by 1.2% and model predictions were different from the observed prevalence by 17.0%.

**Table 3:** Validation analysis of spatial predictions from a Bayesian mixed-effects spatial logistic regression model for trachoma (follicular, TF, or inflammation, TI) in children aged 1–9 years, Southern Sudan.

Group*	Mean error	Mean absolute error	Area under the ROC curve (95% CI)				
			True individual status	True prevalence >0%	True prevalence ≥10%	True prevalence ≥40%	True prevalence ≥70%
1	−0.043	0.139	0.84 (0.83–0.86)	0.95 (0.88–1.00)	0.97 (0.93–1.00)	0.94 (0.87–1.00)	0.93 (0.84–1.00)
2	−0.105	0.192	0.88 (0.85–0.90)	0.94 (0.84–1.00)	0.97 (0.91–1.00)	1.00 (1.00–1.00)	0.86 (0.67–1.00)
3	0.050	0.152	0.78 (0.76–0.81)	0.99 (0.95–1.00)	0.86 (0.65–1.00)	0.91 (0.76–1.00)	0.77 (0.59–0.96)
4	0.045	0.218	0.72 (0.69–0.75)	1.00 (1.00–1.00)	1.00 (1.00–1.00)	0.85 (0.64–1.00)	0.63 (0.40–0.85)
All	−0.012	0.170	0.80 (0.79–0.81)	0.96 (0.93–0.99)	0.96 (0.93–1.00)	0.92 (0.87–0.98)	0.80 (0.72–0.88)

*”Group” refers to four random subsets of locations from the original dataset and “All” refers to the four subsets pooled.

## Discussion

The present study set out to identify areas of Southern Sudan that are of low priority with regards to trachoma control, so that the limited resources available to the National Trachoma Control Program and its implementing partners can be targeted to areas most in need of intervention. Using a Bayesian geostatistical model we determined that prevalence of active trachoma is associated with long-term average rainfall, and that the model containing this variable reliably predicted areas at risk of trachoma transmission. The resulting risk maps show that trachoma control activities need to focus on the centre, north and east of the country, and that large areas in the south-west can, for now, receive a low priority. The predictions were also consistent with prior knowledge of the distribution of trachoma in Southern Sudan. Western Equatoria State, predicted to be of low transmission risk, borders with the Democratic Republic of Congo, which is anecdotally believed to be relatively free from trachoma. Jonglei, Eastern Equatoria and Upper Nile States, in contrast, were predicted to be at risk of high transmission and border parts of Ethiopia, which is known to be highly trachoma endemic [34].

Our findings that older children have a lower prevalence of trachoma than younger children and that an individual's sex is not an important risk factor are consistent with the published literature [7], [8]. Similarly, the finding that rainfall is an important predictor of trachoma transmission in Southern Sudan confirms earlier results of studies from Sudan and Mali, demonstrating that active trachoma was more prevalent in more arid areas [14], [35]. Possible explanations for this observation are that dry conditions: i) might promote trachoma by desiccating the conjunctiva, making it more susceptible to infection, and/or ii) increase the amount of dust particles in the air, hence increasing irritation of the conjunctiva and providing a vehicle for *C. trachomatis* to come into contact with the eye [14]. Access to water may also be limited in dry areas, in turn affecting bodily hygiene measures, such as hand and face washing, hence increasing trachoma transmission by hand-to-eye contact. Lack of water is a known risk factor for trachoma [36], [37]. Additionally, semi-arid areas often tend to be inhabited by seasonally nomadic pastoralists [8] who generally have very low access to sanitation facilities and often defecate in animal pens close to the living areas, hence providing an ideal habitat for the trachoma-transmitting fly *Musca sorbens* in or near their home compounds [38], [39]. It is likely that livelihoods, particularly livestock raising, in addition to other ethnicity-related factors (e.g. house construction methods, isolation of ethnic areas from health centres, and socioeconomic status) are major risk factors for trachoma in Southern Sudan [8].

A limitation of the analysis is the static nature of our model. Seasonal variation in trachoma has been demonstrated [40], but our models did not consider the season in which the data were collected. Substantially more data would be required to predict the spatiotemporal distribution of trachoma in Southern Sudan. A second, clear limitation is the geographical spread of the data, which in some states were obtained from clusters of neighbouring communities, resulting in uneven geographical coverage. This is not surprising given that spatial analysis was not a primary objective of the surveys at the time of their implementation. Uneven geographical coverage of Southern Sudan means that the spatial predictions are more precise, and likely to be more accurate, in areas that are in close proximity to the survey locations, and relatively imprecise and less accurate in areas where there are few data points. While we are less confident of our predictions in some areas compared to others, our analytical approach has the considerable advantage that we can quantify and harness these uncertainties to prioritise future data collection in areas of the country where our predictions are less precise.

The maps developed here can be used, in the first instance, to prioritise surveys aimed at confirming suspected high-risk areas and at generating baseline data to monitor and evaluate subsequent interventions in currently non-targeted areas [28]. The risk maps thus provide a useful complementary tool to trachoma rapid assessments (TRA) and PBPS [41] in that they help to identify areas where collection of additional data would be most useful. Over time, the model presented here can be refined by incorporating new data collected in the identified high risk areas, in turn reducing the uncertainties of the spatial predictions. The findings presented here are in fact the result of multiple iterations, whereby additional data, generated by georeferencing additional sites from previous PBPS were used to revise the spatial models and risk maps. A similar approach could be taken in other countries where some trachoma prevalence data are already available, although this would probably require building of in-country capacity for spatial analysis and/or partnering with international experts. As in Southern Sudan, these data could form the basis for an initial model determining where additional surveys would be most informative. In countries with no or very little trachoma prevalence data it may be advisable to randomly survey individuals (as outlined in the PBPS methodology [31]) in a limited number of locations over a large geographical area, followed by development of a risk map. Suspected high-risk areas can then be targeted with TRAs, followed by PBPS in confirmed endemic areas.

The risk maps also provide a useful tool to target SAFE interventions. The National Trachoma Control Programme in Southern Sudan now has information that allows it to categorize the south-western part of the country as low priority for further surveys, with resources being conserved for central, northern and eastern areas where trachoma is more likely to be endemic. Being able to present these findings in the form of a comprehensive risk map may also make it easier for the MoH-GoSS to engage the broad range of stakeholders that needs to be mobilized to deliver a comprehensive SAFE strategy. Once more data on other NTDs are available, such as schistosomiasis, soil-transmitted helminthiasis and lymphatic filariasis [31], [42], the approach used here can be applied to develop a co-endemicity map that identifies where integrated control of these diseases is warranted [21].

We have demonstrated that trachoma risk mapping, based on integration of field survey and environmental data in statistically robust, spatial statistical models, was achievable and useful in Southern Sudan. Risk mapping is therefore likely to also be applicable to other trachoma endemic settings.
